# Genome Environment Browser (GEB): a dynamic browser for visualising high-throughput experimental data in the context of genome features

**DOI:** 10.1186/1471-2105-9-501

**Published:** 2008-11-27

**Authors:** Derek Huntley, Y Amy Tang, Tatyana B Nesterova, Sarah Butcher, Neil Brockdorff

**Affiliations:** 1 Centre for Bioinformatics, Division of Molecular Biosciences, Imperial College London, London SW7 2AZ, UK; 2 MRC Clinical Sciences Centre, Imperial College Faculty of Medicine, Hammersmith Hospital, Du Cane Road, London W12 ONN, UK; 3 Wellcome Trust Sanger Institute, Wellcome Trust Genome Campus, Hinxton, Cambridge, CB10 1HH, UK; 4 Developmental Epigenetics Group, Department of Biochemistry, University of Oxford, South Parks Road, Oxford OX1 3QU, UK

## Abstract

**Background:**

There is accumulating evidence that the milieu of repeat elements and other non-genic sequence features at a given chromosomal locus, here defined as the genome environment, can play an important role in regulating chromosomal processes such as transcription, replication and recombination. The availability of whole-genome sequences has allowed us to annotate the genome environment of any locus in detail. The development of genome wide experimental analyses of gene expression, chromatin modification and chromatin proteins means that it is now possible to identify potential links between chromosomal processes and the underlying genome environment. There is a need for novel bioinformatic tools that facilitate these studies.

**Results:**

We developed the Genome Environment Browser (GEB) in order to visualise the integration of experimental data from large scale high throughput analyses with repeat sequence features that define the local genome environment. The browser has incorporated dynamic scales adjustable in real-time, which enables scanning of large regions of the genome as well as detailed investigation of local regions on the same page without the need to load new pages. The interface also accommodates a 2-dimensional display of repetitive features which vary substantially in size, such as LINE-1 repeats. Specific queries for preliminary quantitative analysis of genome features can also be formulated, results of which can be exported for further analysis.

**Conclusion:**

The Genome Environment Browser is a versatile program which can be easily adapted for displaying all types of genome data with known genomic coordinates. It is currently available at .

## Background

Common repetitive DNA elements, which include satellite DNA, long interspersed repeats (LINE), short interspersed repeat (SINE) and long terminal repeat (LTR) elements, comprise 37% of the rodent and 42% of the human genome sequence respectively [1,2]. By comparison, exons of genes comprise only approximately 2% of sequence. These common repeat elements, together with other features such as CpG islands [3], scaffold-attachment regions (SARs) [4], and transcription factor binding sites, shape the genome environment in which a gene resides. There is accumulating evidence that the genome environment can be important for the regulation of gene expression. For example, SARs play a role in regulating MHC Class I gene expression in humans [5], LTR retrotransposons influence developmentally regulated expression of genes in mouse oocytes and preimplantation embryos [6], and LINE-1 (L1) elements modulate transcription of human genes [7].

With the DNA sequence data generated from genome projects, we can now paint a fuller picture of a gene's environment *in silico*. Added to this, the development of high throughput DNA sequence-based experimental strategies such as whole-genome gene expression microarrays and ChIP-on-chip/ChIP sequencing means that it is now possible to look for correlations between underlying sequence features, the transcriptome, and epigenetic features such as DNA methylation, covalent histone modification and chromatin protein distribution. Importantly, novel bioinformatics and software tools are needed, both to analyse the large datasets generated by such studies and to facilitate elucidation of previously unappreciated relationships between underlying sequence features, gene expression and epigenetic modification. Here we describe development of the Genome Environment Browser, a novel tool to aid visualisation and analysis of genome wide data in the context of underlying genomic features.

## Implementation

GEB is designed as a set of software components that automatically build a core database of genomic feature data from the Ensembl database for any available species, using the Ensembl Perl API, with the features to be retrieved defined in a configuration file. The settings for the local storage database and Ensembl connection are also stored in the configuration file so once initialized the software automatically builds the GEB data without the requirement for further user input. For repeat features, such as LINEs, individual classes of the repeat can be defined to be stored separately to view as an individual track in the GEB viewer. We have used this feature for the display of LINE L1 elements. The data is stored in a standard relational database, specifically MySQL [8]. Alternatively we provide pre-built databases of the latest Ensembl builds for human, mouse and rat on our web site. These can be used as the basis of a core GEB installation to which users' own data can be added. Further scripts are provided for the storage of non-Ensembl features and microarray data, both expression and ChIP-chip. These scripts require the data to be in a tab delimited format, which can be created for example by parsing genomic annotation software output or from an Excel spreadsheet for microarray data. We have used this feature for the LINE L1 components (UTRs and ORFs) and CpG island predictions within our custom annotations. We found the CpG island Ensembl predictions to be conservative so for our predictions we chose to use the EMBOSS newcpgreport program [9], the output of which was parsed to produce a tab delimited file as required. To facilitate the ease of adding data to GEB, including the core database, a Java graphical user interface is provided to manage the uploading of the database itself and addition/deletion of other feature data, including microarray data. This program can be used in place of the Perl scripts but it is platform-dependent and is only suitable for Windows users. See additional file 1 for the full GEB installation guide.

A separate, platform-independent, standalone Java front-end application provides a graphical interface for navigating the database and visualisation of the genome features. The Java application was created using the GenoViz SDK [10], a Java toolkit providing custom classes for the visualisation of genomic annotation data.

## Results and discussion

### Genomic feature annotation and browsing

We have been investigating a proposed role for the L1 retrotransposon in facilitating X-chromosome inactivation, the process whereby a single X chromosome is epigenetically inactivated in cells of female mammals [11]. L1s (a major family of LINEs) are non-LTR retrotransposons 6–7 kb in length that collectively account for approximately a fifth of the mammalian genome [12]. To facilitate this study we have developed a novel software tool, the Genome Environment Browser (GEB), for visualising epigenetic and transcriptomic features in the context of the genome environment.

**Figure 1:**
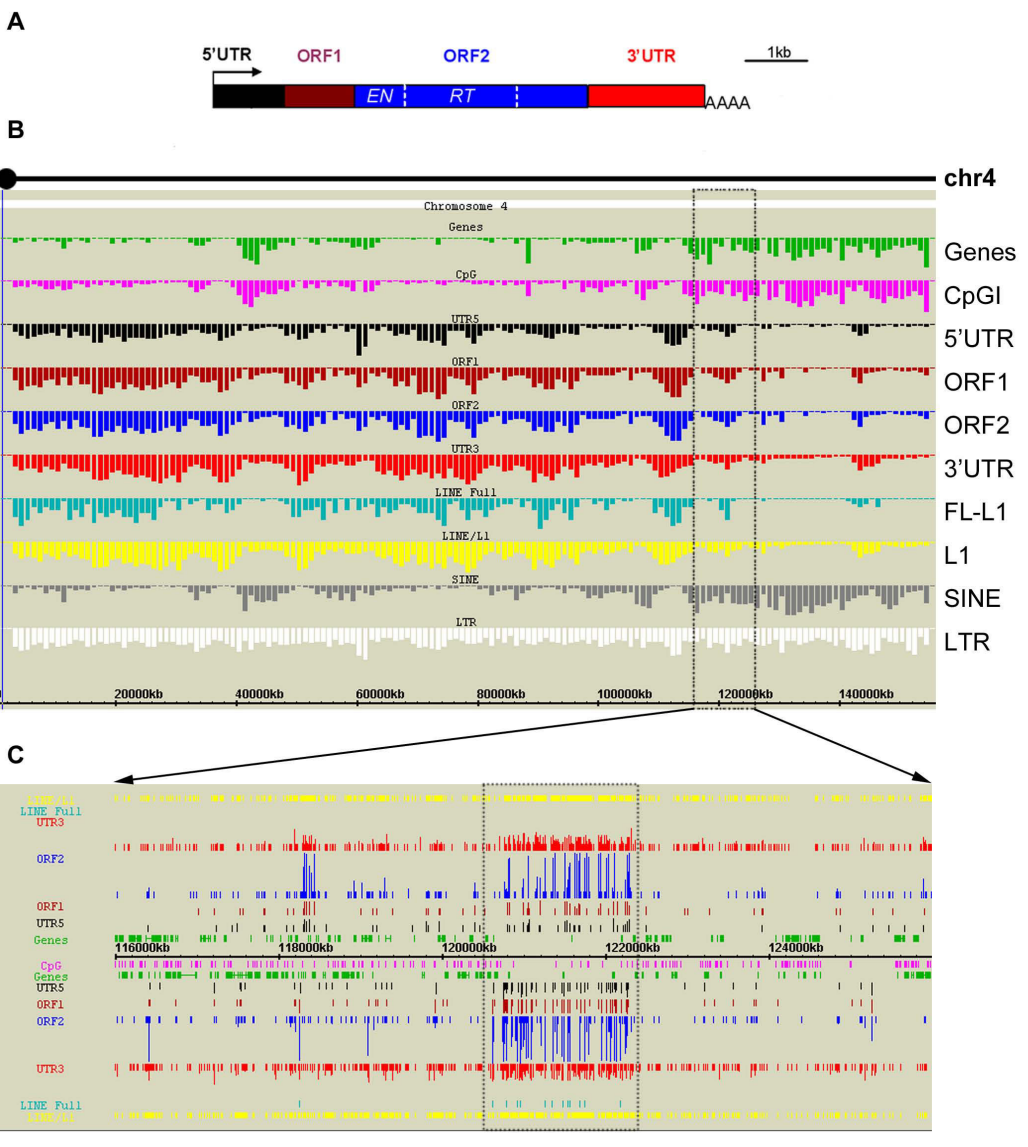
**GEB histogram and physical map displays**. (A) The general structure of LINE-1 (L1) elements in mammals. The arrow indicates internal promoter for transcription. Between the 5' and 3' untranslated regions (UTRs) are two open reading frames (ORFs) which encode proteins essential for autonomous retrotransposition. *EN*, endonuclease domain; *RT*, reverse transcriptase domain; AAAA, polyadenylation site. (B) GEB histogram display for mouse Chromosome 4 from centromere to telomere (left to right). The width of each bar represents 1 Mb, and the height of each bar represents the frequency of defined features. (C) GEB physical map display of a 10 Mb region (116–126 Mb), corresponding to the boxed area in (B). Features on the forward and reverse strands are shown above and below the ruler respectively. The power of the two-dimensional display is illustrated by the custom annotation of L1 elements, where each copy varies substantially in size. With the physical length of each individual L1 element plotted on the horizontal axis and the homology of each L1 component to the L1 consensus plotted on the vertical axis, regions with high L1 density (dashed box) are clearly distinguished from relatively L1-poor regions on visual inspection (compare the 5'UTR-ORF1-ORF2–3'UTR plot against the yellow LINE/L1).

Currently GEB is configured to display Human and Mouse genomes. While many public genome browsers are designed to be gene-oriented, GEB focuses on both genes and non-genic elements in their vicinity. In the default version of the browser, CpG islands, LINEs (including families LINE-1, LINE-2 and LINE-3), L1s, SINEs and LTRs are annotated in detail in the context of known Ensembl genes and non-coding genes (mainly functional RNAs). Because of our interest in L1 elements we have included annotation of the functional components, the 5' UTR, ORF1, ORF2 and 3' UTR (Figure 1A), and additionally full length (FL) L1s. The distribution and density of all these features can be visualised at two levels: first a histogram display for a panoramic, whole-chromosome view (Figure 1B), and second, a physical map display for more detailed analysis of regions between 500 bp and 25 Mb (Figure 1C). See additional file 2 for the GEB user guide.

The histogram display is composed of a parallel set of histograms, each plotting the copy number/density of a genome feature across the entire chromosome (typically per Mb). It is powerful in capturing the modularity of genome features across a chromosome. For example, the previously reported positive correlation between CpGI and genes [13] and the reciprocal relationship between L1 and SINEs [14,15] are reproduced graphically (Figure 1B). Specific regions with interesting patterns, e.g. a gene-rich region adjacent to a gene desert, can be easily identified and precisely selected on the histogram display to be examined in greater detail in the physical map display.

In the physical map display, unlike some public genome browsers, all genome features (including interspersed repeats) are displayed on their respective strands. A very important feature of GEB is the capability to display genomic features in two-dimensional multi-colour graphics reminiscent of a dot-plot, as illustrated by our custom annotation of L1 elements. The length of homology of an L1 element to each functional component in the FL-L1 consensus sequence is plotted on the vertical axis, whereas the physical length of the L1 element is indicated on the horizontal axis as in public genome browsers (Figure 1C). Relatively rare FL-L1s appear as a long, continuous diagonal arrangement of UTRs and ORFs, and are clearly distinguished from the bulk of truncated L1s (mostly remnants of the 3' UTR), shown as shortened lines or bars.

Another key feature of GEB is the rapid transition between high and low resolutions in the physical map display. The "condensed" view of the selected region is initially displayed at 10–200 kb resolution (depending on the size of the region), and can be zoomed in on the same screen flexibly to a detailed 2 bp resolution (and zoomed back out again), abolishing the need to refresh or load a new page. This allows uninterrupted visual scanning of genome features and pattern searching across very large genomic distances, up to 25 Mb. In essence, the physical map display provides a zoomed-in view of the spatial relationship between features across the region of interest. Local enrichment or depletion of any type of feature can be readily visualised.

### Data retrieval and preliminary quantitative data analysis tools

**Figure 2:**
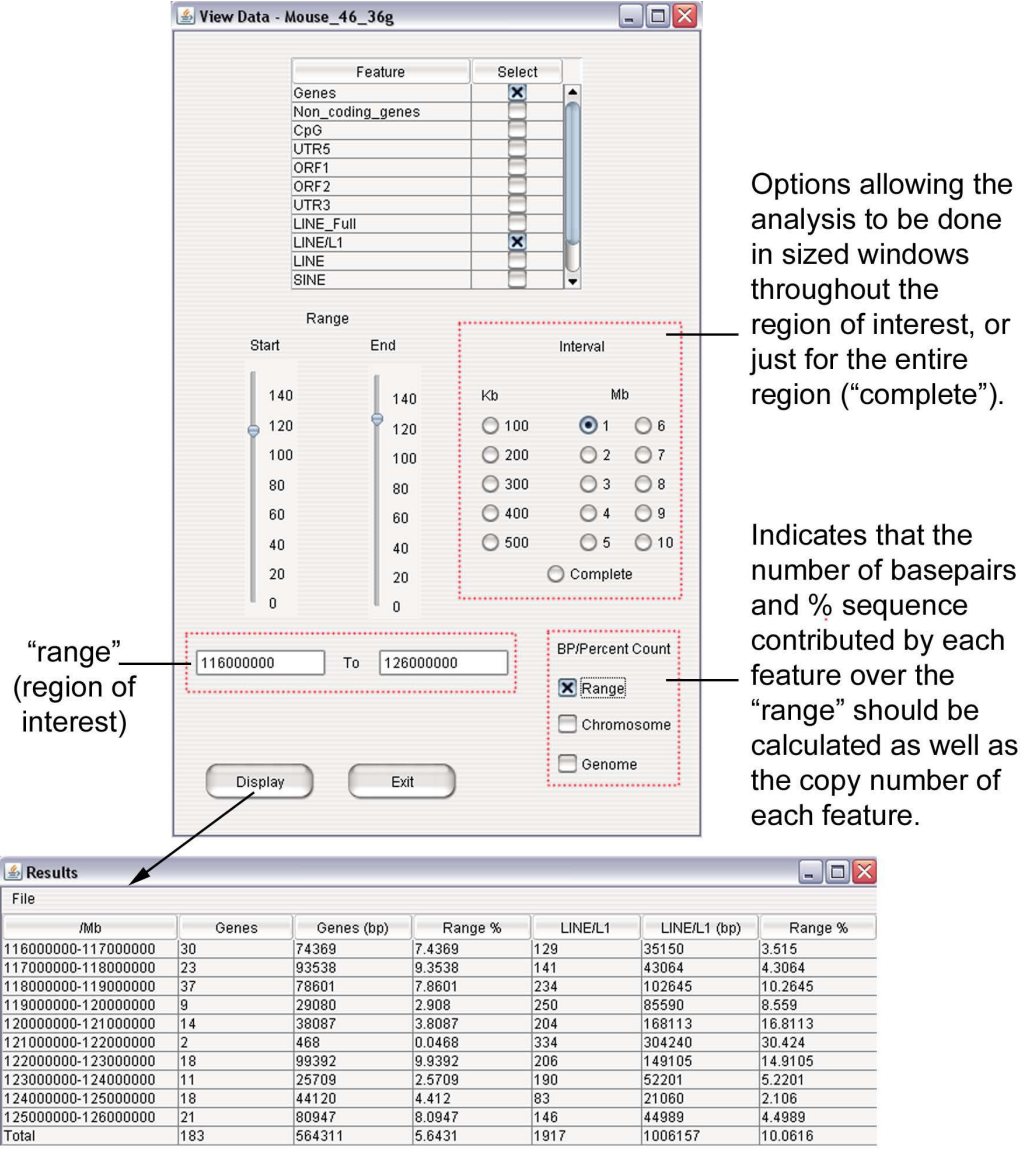
**ViewData for quantitative analysis of genome features**. Analysis of gene and L1 content in the 10 Mb region of mouse chromosome 4 shown in Figure 1C. Shown here is a sample query to calculate the copy number and percentage sequence of LINE-1 and genes in ten 1 Mb windows. Percentage sequence data were calculated per window and for the entire 10 Mb region. The results table can be exported and saved as a tab-delimited file for further analyses.

Detailed textual annotation information on all features can be explored at a mouse click in physical map display. For the retrieval of other types of annotation data (e.g. gene ontology, protein-related annotation, homology to genes in other species), we have incorporated a functionality in GEB which brings up relevant Ensembl pages (ContigView and GeneView respectively) in the user's default web browser, depending on whether a genomic region or a gene has been selected in GEB histogram/physical map displays. Additionally, a simple quantitative sequence analysis tool is available, which calculates the copy number and/or percentage sequence representation of any type of annotated feature across a genome region of any size. For a more detailed analysis, the calculation can also be done in defined windows (e.g. every 100 kb) across a region (Figure 2).

### Displaying custom experimental data in GEB

**Figure 3:**
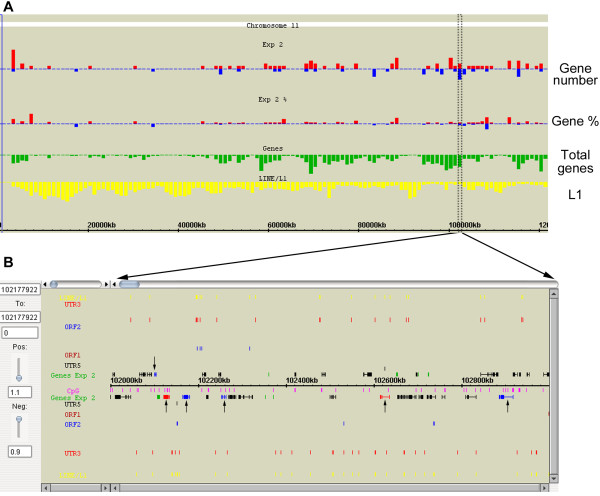
**GEB displays for gene expression microarray data**. (A) Histograms showing demonstration data on the distribution of differentially expressed genes across mouse chromosome 11. The width of each bar is 1 Mb. Red and blue bars correspond to upregulated and downregulated genes respectively. The "gene number" panel shows the number of DEGs in a given Mb, while the "gene %" panel shows the percentage of DEGs out of all genes in that Mb. (B) Physical map display showing DEGs (indicated by arrows) from a 1 Mb region (102–103 Mb) in more detail. Genes are colour coded depending on whether they are upregulated (red), downregulated (blue), or unchanged in expression (black). Genes with no data in expression appear green (default colour for physical map displays). Stringency of gene expression thresholds for DEGs can be adjusted in real-time using the sliding bars on the left: "Pos" (positive) for upregulated genes, "Neg" (negative) for downregulated genes. The colour of genes will change accordingly.

The GEB histogram and physical map display framework can be applied to one or more sets of custom genome/chromosome-wide experimental data in the context of genome environment, for instance, measurements of gene expression, location of transcription factor binding sites/DNA sequence motifs, ChIP-on-chip/ChIP-seq mapping of histone tail and CpG methylation modifications. Gene expression array data can be presented in two complementary ways in the histogram display (Figure 3A). First, the number of differentially expressed genes (DEGs) (up- or downregulated) per Mb interval can be plotted alongside other genome features of interest. Second, taking into account some regions are inherently more gene-rich than others, GEB also allows plotting in each window the proportion of DEGs normalised to the underlying gene density. Specific regions of interest can be explored in detail, again in the physical map display, with an added functionality where genes are colour-coded to reflect their expression status (downregulated, upregulated, or no change/data unavailable). Additionally, the cut-offs/thresholds defining differential expression can be altered in real-time using an incorporated slide-bar, allowing the user to observe how the pattern of DEG distribution changes (Figure 3B).

**Figure 4:**
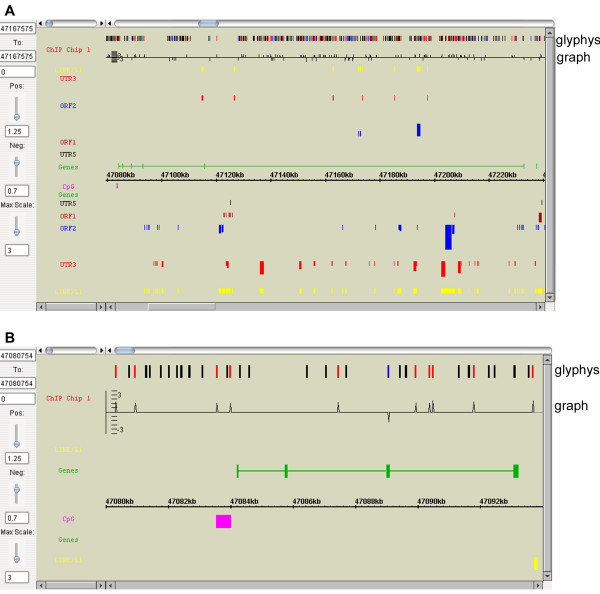
**GEB displays for tiling microarray data**. (A) Physical map display of demonstration tiling array data for a ~160 kb region on mouse chromosome 18 covering the *Commd10 *gene. Each colour-coded glyph corresponds to one tiling probe in the region, where the colour reflects enrichment (red), depletion (blue) or no change (black) of signal relative to the control. Closely-packed glyphs may generate blocks of red, blue or black, allowing users to quickly scan across a region for specific patterns. As for expression array data display, the enrichment/depletion thresholds can be adjusted in real-time using the sliding bars on the left, and the colours of the glyphs will change accordingly. Additionally, the maximum scale ("Max scale") for the y-axis of the graph can also be adjusted in real-time. (B) A 14 kb physical map zoomed in from Figure 4A, showing the first four exons of the *Commd10 *gene. As the glyphs (probes) and the graph are less condensed, the peaks and troughs of the graph can be seen clearly, allowing users to study local patterns in detail.

GEB's flexible and efficient browsing capabilities are pivotal in the visualisation of any patterns embedded in densely-tiled probes over long genomic distances, as in the case of ChIP-on-chip data. The interface for displaying data from tiling arrays shares many of the features described above for gene expression data, with two specific features in the physical map display accommodating for the higher probe density. A "glyphs" track has been designed for appreciating global patterns, where all or a subset of probes can be colour-coded according to their ChIP-enrichment levels/status and displayed linearly (as if the probes were individual genes) (Figure 4A). To discern local patterns, the exact ChIP-enrichment levels for each probe can also be plotted in the "graphs" track with an adjustable scale for the Y-axis (Figure 4B).

### Comparison of GEB with other genome browsers

Relative to major public genome browsers (e.g. Ensembl Genome Browser [16], UCSC Genome Browser [17] and NCBI Map Viewer [18]), GEB provides novel visualisation tools to analyse patterns of genome features and experimental data along an entire chromosome, and additionally the capability to move rapidly to a scalable detailed view with a dynamic range between 25 megabases and 2 basepairs resolution. Thus, users can move seamlessly through data covering a large genomic region, for example zooming in and out from megabase scale gene clusters to specific genes, without the need to change to a new page. Additionally GEB has relatively detailed annotation of interspersed repeats, including directionality, and provides simple tools to quantify the density/frequency of a given genomic feature within user defined limits. The latter are important for preliminary quantitative data analysis in cases where interesting patterns have been visualised.

GEB also has unique features relative to more recently developed track-based public browsers for integrating genome and experimental data, such as Argo Genome Browser [19] and Integrative Genomics Viewer [20], notably the detailed annotation of dispersed repeats and unique display options of GEB, the use of the anti-parallel "top and bottom" strand representation (as used in Ensembl) for detecting potential strand-bias in the distribution of selected features (e.g. gene clusters), the interactive visualisation tools for chromosome-wide feature density/distribution, and the quantitative data analysis tools.

## Conclusion

GEB offers a number of unique and novel features and as such complements existing tools for use in studies relating whole genome scale experimental data with underlying genome features. The software is currently available at . The program is easily adaptable for viewing all kinds of data in the context of genome environment from different sequenced genomes, provided the chromosomal start and end positions for each feature are known, e.g. data from ChIP-sequencing and chromosome conformation capture. The two-dimensional display used for L1 data in our example is potentially useful for other repetitive features which vary substantially in size. A complete set of GEB documentation as well as files for installing and running GEB is provided in additional file 3.

## Availability and requirements

• **Project name: **GEB

• **Project home page: **

• **Operating system(s): **Unix/Linux for data retrieval and processing, platform independent for MySQL database and Java visualisation software

• **Programming language: **Perl and Java

• **Other requirements: **Java 1.6+, Perl, including Ensembl Perl API, and MySQL

• **License: **GNU GPL

• **Any restrictions to use by non-academics: **None

## Abbreviations

GEB: Genome Environment Browser; CpGI: CpG island; LINE: long interspersed nuclear element; SINE: short interspersed nuclear element; LTR: long terminal repeat; L1: LINE-1; FL: full-length; UTR: untranslated region; ORF: open reading frame; ChIP: chromatin immunoprecipitation; DEG: differentially expressed genes.

## Authors' contributions

DH designed the software, wrote the source code and drafted the manuscript. YAT conceived of the study, designed the software and drafted the manuscript. NB and TBN conceived of the study, participated in its design and coordination, and edited the manuscript. SB coordinated the study. All authors read and approved the final manuscript.

## Supplementary Material

Additional file 1 GEB installation guide. Detailed documentation on installing GEB. Click here for file

Additional file 2 GEB user guide. GEB tutorial with detailed description of each feature and functionality of the browser. Click here for file

Additional file 3 An archive of files for the GEB Java display, GEB set up and documentation. This includes all the files mentioned in the manuscript and GEB installation guide, e.g. the Java display file (GEB.jar), the sample configuration file (GEB.ini) and the Perl scripts/modules required to build the dedicated GEB database.
Additional file 3 is a zipped archive file. We recommend using gunzip on the Mac (OSX) and Unix/Linux, and WinZip or WinRAR on Windows to open the file. Click here for file
