# Supplementary material for: Genome Environment Browser (GEB): a dynamic browser for visualising high-throughput experimental data in the context of genome features
Source: BMC Bioinformatics. 2008 Nov 27;9:501. doi: 10.1186/1471-2105-9-501 (PMC2614995; doi:10.1186/1471-2105-9-501)
Supplement: Additional file 1 — GEB installation guide. Detailed documentation on installing GEB. [file 1471-2105-9-501-S1.pdf]

Genome Environment Browser (GEB) installation guide

Creating the GEB Database

The core of GEB relies on the Ensembl database and there are 2 options to make this data available to your installation of GEB. The simplest option is to download core databases provided on the GEB web site (web.bioinformatics.ic.ac.uk/geb). At present we provide the latest Ensembl builds for human, mouse and rat. It would probably be possible to add other core databases, including different versions, if requested. The only other requirement is a local copy of the MySQL database. This can be downloaded free from www.mysql.com.

Note: when installing MySQL ensure that the option to include the "bin" directory in your Windows PATH is selected.

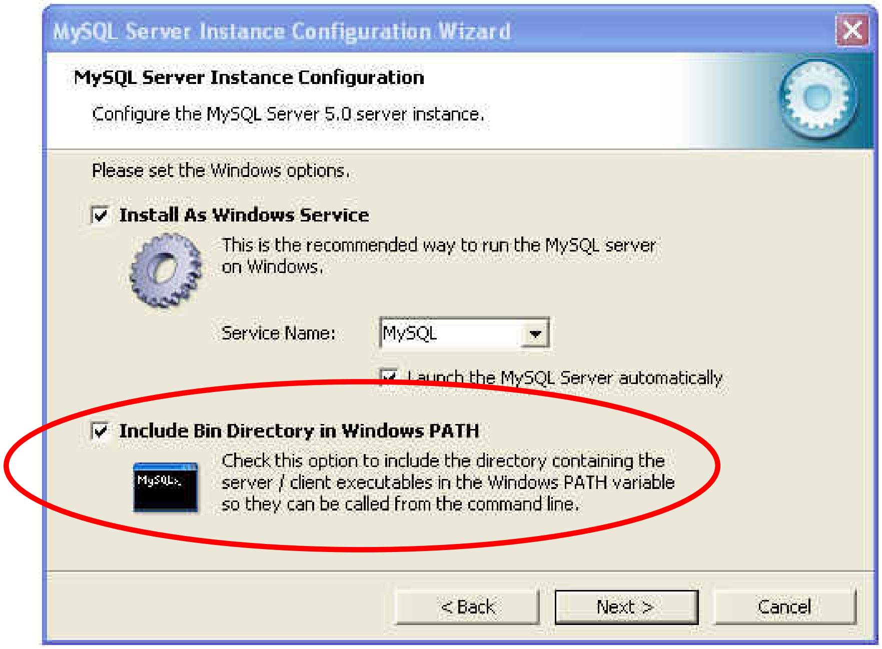

WINDOWS USERS - To install the downloaded database, add extra feature data, including microarray data, and manage existing installations the GEB_Setup.jar file is used. This is launched by double clicking the GEB_Setup.bat file, which defines system settings required for the program. Double click the file to launch it. The setup program requires access to the geb.ini file, which must have the database settings set for you version of MySQL. See **(II) Configurating and launching GEB** in the GEB user guide.

UNIX/LINX and MAC USERS – The database dump downloaded from the web site will need to be loaded into the database from the command line. It is a standard

database dump generated by mysqldump. The addition of other feature and microarray data can be achieved with the GUI, which should be launched from the command line by typing:

```
java -Xms512m -Xmx1024m -cp GEB_Setup.jar GEB_Setup.GEB_Setup_GUI
```

Alternatively, feature and microarray data can be added with the Perl scripts provided in the Perl directory:

To load feature data the Perl script can be used as detailed in section (IV) - Adding non-Ensembl Features.

To load microarray data the Perl scripts can be used as detailed in section (V) - Adding Microarray Data.

Database Installation

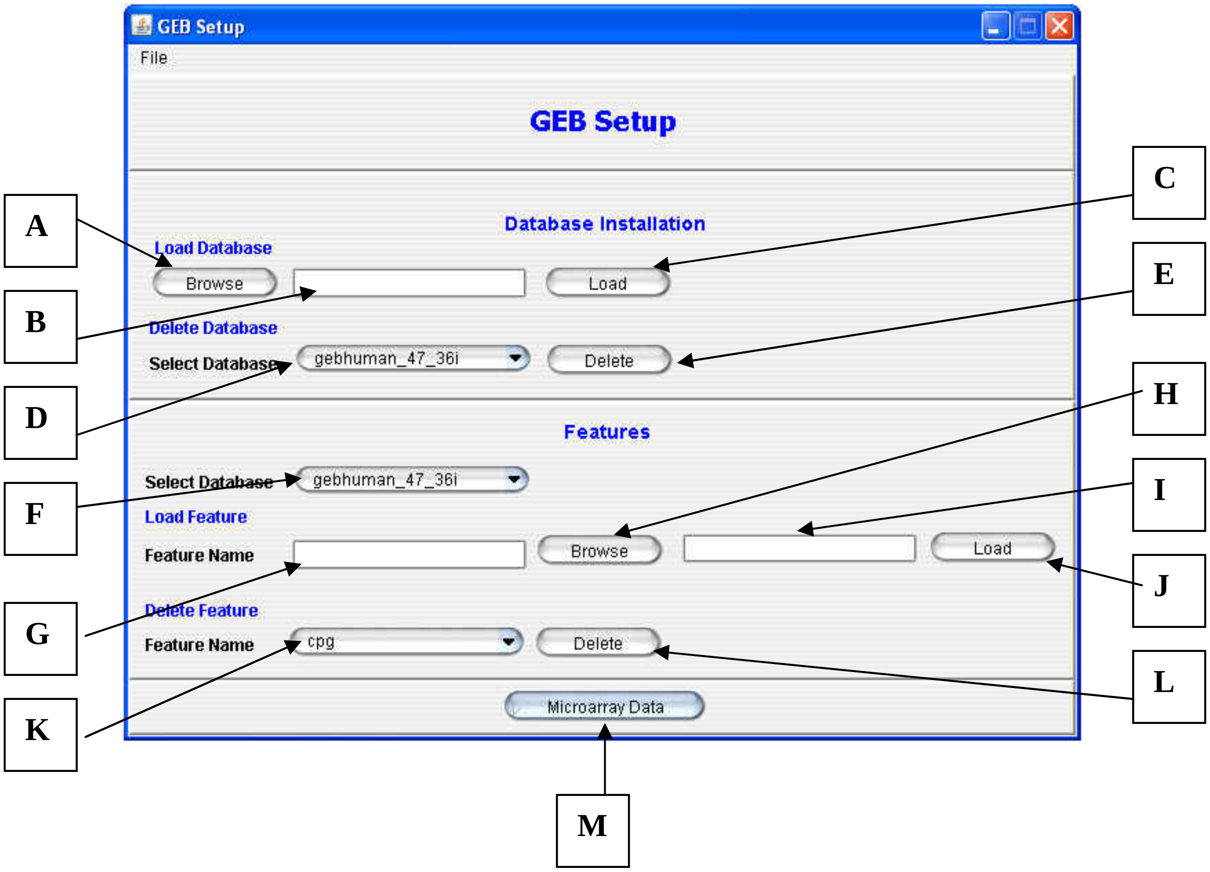

A – Browse for a database file downloaded from the GEB web site.

B – The database file will be displayed here.

C – Load the database file into your MySQL database. This may take a few minutes.

D – Drop down list show currently installed GEB databases. These can be selected for deletion from the MySQL database.

E – Delete the selected GEB database. A warning is shown prior to the database being deleted.

Features

This section is used to add/delete features to a database. Any genomic feature data can be added to GEB, as long as it has chromosome start and end positions. The data needs to be in a tab-delimited text file where the columns are:

Chromosome (For the X or Y chromosome the integer value is required)
Chromosome start
Chromosome end
Description

The description is a free text field. For the local installation of GEB the CpG data, predicted by newcpgreport, the description is created to include the size, sum, percentageGC content and observed/expected value of the prediction. For example:

Size: 242 Sum: 128 PC GC: 52.89 Obs: 0.83

F – Select the GEB database where features are to be added/deleted.

G – Enter a suitable name for the feature. This will be used a label for the associated track on the GEB display.

H – Browse for the file containing the feature data, correctly formatted as detailed above.

I – The file is displayed here.

J – Load the feature data into the database.

K – Select a feature to be deleted from the chosen database (**F**).

L – Delete the feature from the database. A warning is shown prior to the feature being deleted.

M – Launch the microarray data setup display.

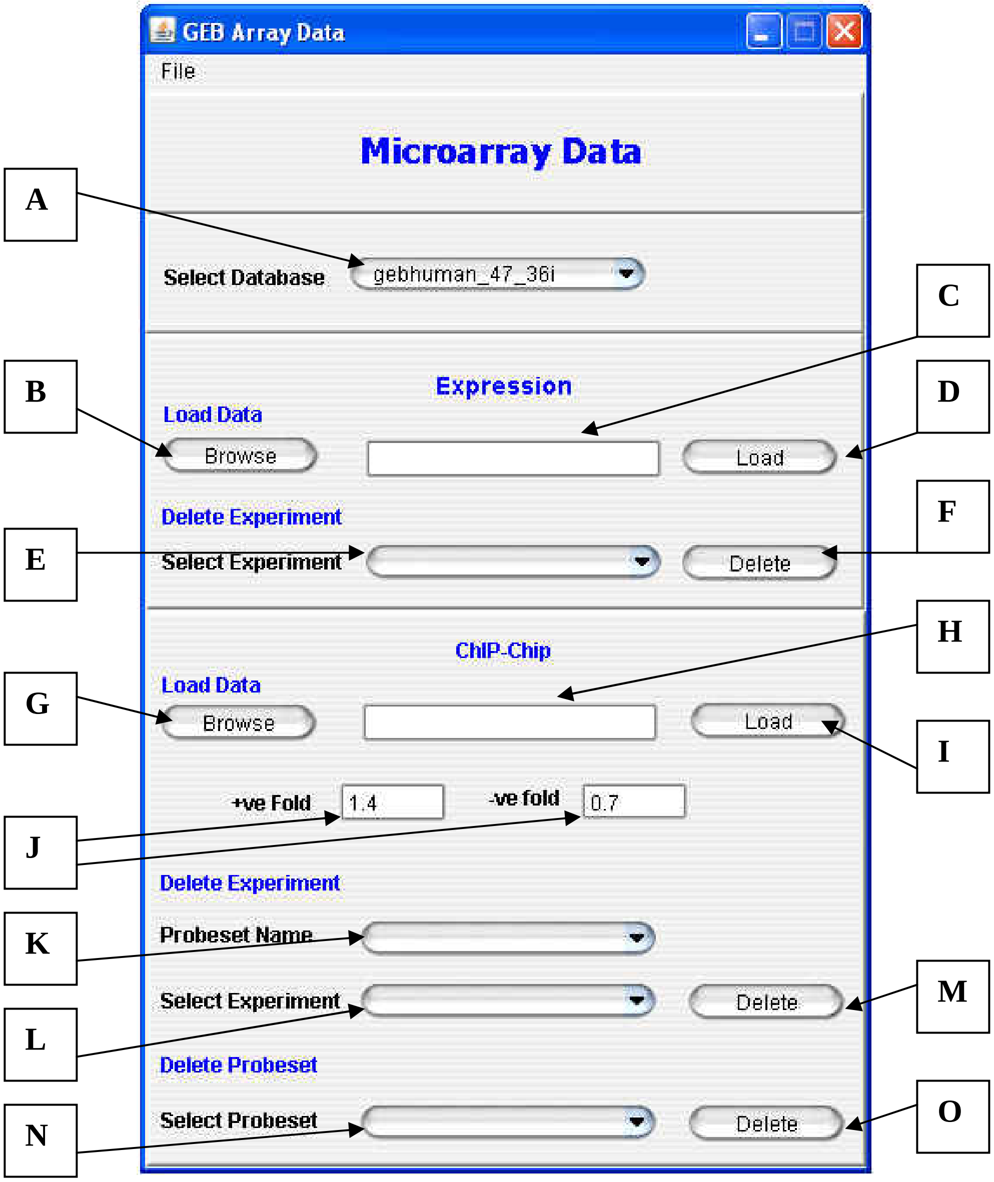

A – Select the GEB database where microarray data is to be added/deleted.

Expression

The expression array data needs to be in a tab-delimited text file where the columns are:

Chromosome (For the X or Y chromosome the integer value is required)

Ensgene ID The ensgene id for the gene

Experiment A name to identify the experiment. Several experiments can be stored so a unique name is required for each one

Expression The expression value for this gene

An example row is:

1	ENSMUSG00000014602	Experiment_1	0.786

B – Browse for the file containing the expression microarray data, correctly formatted as detailed above.

C – The file is displayed here.

D – Load the microarray data into the database.

E – Select an experiment to be deleted from the chosen database (**A**).

F – Delete the experiment from the database. A warning is shown prior to the experiment being deleted.

ChIP-Chip

ChIP-Chip array data is handled slightly differently. The main difference being that the data for the histogram display is dynamically generated for the expression arrays, based on selected expression value cut off, but pre-calculated for the ChIP-Chip probes. This is necessary due to the potentially large numbers of ChIP-Chip probes that make dynamic calculation too slow.

As with the expression array data, the ChIP-Chip data needs to be in a tab-delimited text file where the columns are:

Chromosome (For the X or Y chromosome the integer value is required)

Probeset This is a name for the probeset used. Different experiments can use the same probeset and have the same probeset name.

Experiment A name to identify the experiment. Several experiments can be stored so a unique name is required for each one.

Probe The probe id

Chromosome start

Chromosome end

Expression The expression value for this probe

An example row is:

3	Nimblegen	Exp_2	XF-80-100	96097632	96097691	1.16668

G – Browse for the file containing the ChIP-Chip microarray data, correctly formatted as detailed above.

H – The file is displayed here.

I – Load the microarray data into the database.

J – The cut-off expression value for a probe to be included in the histogram display.

K – To delete an experiment from the database the probeset name is first selected.

L – Select an experiment to be deleted from the chosen database (**A**).

M – Delete the experiment from the database. A warning is shown prior to the experiment being deleted.

N – Select an experiment to be deleted from the chosen database (**A**). This will delete all of the associated experiements.

O – Delete the probeset from the database. A warning is shown prior to the probeset being deleted.

Once the GEB database has been created the main GEB display can be launched.

Running the GEB Java Display

The GEB Java program is a self executable jar file that can be launched by double clicking at the **GEB.jar** file, or on the command line by typing:

java –jar GEB.jar

See the user guide for instructions on using GEB.

Manual Preparation of GEB Databases

An alternative to using the Java interface is to use the Perl scripts provided in the Perl directory to build a GEB database of any species/version available in Ensembl. The instructions for using this method are below.

(I) GEB Requirements

The core of GEB relies on the Ensembl database and so requires the Ensembl Perl API. Full installation instructions for this can be found on the Ensembl web site at http://www.ensembl.org/info/using/api/api_installation.html. A new API is released with each version of Ensembl so the correct version should be used for the required Ensembl build. Note that the version of BioPerl on this page is also required.

Other Perl module requirements are:

Config::IniFiles
DBI
DBD::mysql

GEB also requires write access to a MySQL database.

The scripts provided assume Perl is installed under /usr/local/bin/perl. If this is not the case then the following scripts will need to be changed on the first line for your installation of Perl:

initialiseGEB.pl
loadFeature.pl
loadArrayData.pl
createChipArrayTotal.pl

(II) GEB Configuration

The installation of the core of GEB is handled by a script that reads a configuration file - geb_initialise.ini. This file needs to be edited for your installation of GEB. It defines the database settings and the Ensembl and other features to be stored. The installation script will then create all of the necessary tables and download and store all of the required Ensembl data. Non Ensembl data, such as microarray data, is handled separately.

The provided geb_initialise.ini file can be used as a template.

Note: All settings, except defined features and repeats, are in lower case.

The required sections are:

> ***[database]***
> ***host = localhost***
> ***port = 3306***
> ***username = guest***
> ***password = guest***

This defines the settings for the local MySQL database. A host, username and password are required and the port only needs to be changed if it is not the default (3306).

Each species to be stored has its own section in the file, starting with the species name section:

> ***[human]***

The settings in each species name section are:

> ***create = yes***
> ***new_db = yes***
> ***name = homo_sapiens***
> ***version = 46_36h***
> ***chromosomes = 24***
> ***x = 23***
> ***y = 24***

- ***create*** – determines if Ensembl data for this species will be downloaded. A configuration file can be created for multiple species and each downloaded in turn by setting this to ***yes*** and all others to ***no***.

- ***new_db*** – if set to ***yes*** this will create a new instance of the GEB database for this species, deleting any previous versions. If set to ***no*** an existing database will be expected and any data will be deleted.

- ***name*** – this is the Ensembl species name.

- ***version*** – this is the Ensembl version number. The GEB database created will be the species and version number so it is possible to have separate versions of the database for the same species but different version numbers if required.

- ***chromosomes*** – the number of chromosomes this species has.

- **x** – the X chromosome number.
- **y** – the Y chromosome number.

After the initial species settings the individual features to be downloaded are defined, starting with the general features:

[features_human]

Any features with a fixed chromosome start and end position that are accessible by the Ensembl API can be downloaded. Defaults are:

Genes = yes
Gon_coding_genes = yes
CpG = yes

- ***Genes*** – if set to ***yes***, protein-coding genes will be downloaded from Ensembl, processed and stored.
- ***Non_coding_genes*** – if set to ***yes***, non-coding genes will be downloaded from Ensembl, processed and stored. If set to ***no*** (or omitted entirely) and ***genes*** is set to ***yes*** then non-coding genes will be included with the main gene download.
- **CpG** – if set to ***yes*** the Ensembl CpG island predictions will be downloaded. (**Note:** the Ensembl CpG predictions are fairly restrictive and we have found they often miss genuine islands. For our local version we generate our own predictions using the EMBOSS program, newcpgreport. These predictions are then added to GEB using the method for non-Ensembl feature data detailed later.)

[repeats_human]

Repeats are handled separately from other general features and there are several options for downloading them. They can either be filtered based on their type (LINE, SINE, etc), their class within a type (LINE L1 elements, for example) or any that are not individually filtered can be grouped together. Defaults are:

LINE/L1 = yes
LINE = yes
SINE = yes
LTR = yes
Other_repeats = yes

- ***LINE/L1*** – if set to ***yes***, LINE L1 elements will be filtered from the LINE repeats and stored separately. This is included only to demonstrate how repeats of a particular class can be filtered. Any individual class of repeats required needs to be listed before the main repeat, in this case LINE.

- ***LINE*** – if set to ***yes***, any LINE elements that have not been previously filtered (in this case L1) are downloaded and stored.

- ***SINE*** – if set to ***yes***, SINE elements are downloaded and stored.

- ***LTR*** – if set to ***yes***, LTR elements are downloaded and stored.

- ***Other_repeats*** – if set to ***yes***, any other repeats, not previously filtered, will be downloaded and stored together.

[microarray_human]

The last option in the configuration file is for microarray data, if it is required. These settings will simply create the database tables for storing the microarray data.

- ***expression*** – should be set to ***yes*** if expression microarray data is to be stored. Otherwise can be set to ***no***, or omitted entirely.

- ***chip_chip*** – should be set to ***yes*** if ChIP-Chip microarray data is to be stored. Otherwise can be set to ***no***, or omitted entirely.

(III) GEB Installation

When the configuration file is complete the creation of the GEB database(s) and downloading and installation of the required Ensembl data is implemented by simple running the ***initialiseGEB.pl***:

```
./initialiseGEB.pl   (if the script has been made executable) or
perl initialiseGEB.pl
```

The configuration script can have any name but the initialiseGEB.pl script expects it to be geb_initialise.ini by default. If you use a file with a different name it needs to be specified when the initialisation script is run:

```
./initialiseGEB.pl  -c  config_file_name
```

Depending on your network connection etc the installation of the data for each required species may take a few hours.

Once completed the Java viewer can be run to visualise the downloaded data.

(IV) Adding non-Ensembl Features

Any genomic feature data can be added to GEB, as long as it has chromosome start and end positions. The data needs to be in a tab-delimited text file where the columns are:

Chromosome (For the X or Y chromosome the integer value is required)
Chromosome start
Chromosome end
Description

The description is a free text field. For the local installation of GEB the CpG data, predicted by newcpgreport, the description is created to include the size, sum, percentageGC content and observed/expected value of the prediction. For example:

Size: 242 Sum: 128 PC GC: 52.89 Obs: 0.83

The feature data file is loaded into GEB using the ***loadFeature.pl*** script. The script takes several arguments, some required and one optional. The arguments are:

-c (Optional) If the configuration file is not the default geb_initialise.ini it needs to be declared. The file is required for the database settings, etc.

-s (Required) The species this feature is for (Human, Mouse etc)

-i (Required) The file containing the feature data

-f (Required) The feature name. This will be used for the feature label in the Java display

Run the script, with the required arguments, and the file will be processed and loaded into the database for visualisation in the Java display.

(V) Adding Microarray Data

Both expression and ChIP-Chip microarray data can be displayed by GEB but they are handled slightly differently.

(V.I) Expression Array Data

The expression array data needs to be in a tab-delimited text file where the columns are:

Chromosome (For the X or Y chromosome the integer value is required)

Ensgene ID The ensgene id for the gene

Experiment A name to identify the experiment. Several experiments can be stored so a unique name is required for each one

Expression The expression value for this gene

An example row is:

1 ENSMUSG00000014602 Experiment_1 0.786

The expression array data file is loaded into GEB using the ***loadArrayData.pl*** script. The script takes several arguments, some required and one optional. The arguments are:

-c (Optional) If the configuration file is not the default geb_initialise.ini it needs to be declared. The file is required for the database settings, etc.

-s (Required) The species the array data is for (Human, Mouse etc)

-i (Required) The file containing the expression array data

-t (Required) The table to load the data into. In this case it is "***Expression***".

Run the script, with the required arguments, and the file will be processed and loaded into the database for visualisation in the Java display.

(V.II) ChIP-Chip Array Data

ChIP-Chip array data is handled slightly differently. The main difference being that the data for the histogram display is dynamically generated for the expression arrays, based on selected expression value cut off, but pre-calculated for the ChIP-Chip probes. This is necessary due to the potentially large numbers of ChIP-Chip probes that make dynamic calculation too slow.

As with the expression array data, the ChIP-Chip data needs to be in a tab-delimited text file where the columns are:

Chromosome (For the X or Y chromosome the integer value is required)

Probeset This is a name for the probeset used. Different experiments can use the same probeset and have the same probeset name.

Experiment A name to identify the experiment. Several experiments can be stored so a unique name is required for each one.

Probe The probe id

Chromosome start

Chromosome end

Expression The expression value for this probe

An example row is:

3 Nimblegen Exp_2 XF-80-100 96097632 96097691 1.16668

As with the expression array data, the ChIP-Chip array data file is loaded in to GEB using the ***loadArrayData.pl*** script. The script takes the same arguments as for expression arrays. The arguments are:

-c (Optional) If the configuration file is not the default geb_initialise.ini it needs to be declared. The file is required for the database settings, etc.

-s (Required) The species the array data is for (Human, Mouse etc)

-i (Required) The file containing the expression array data

-t (Required) The table to load the data into. In this case it is “***ChIP_Chip***”.

Run the script, with the required arguments, and the file will be processed and loaded into the database for visualisation in the Java display.

ChIP-Chip expression array data requires further processing to generate the histogram data for the Java display. When the ChIP-Chip data is loaded, the ***createChipArrayTotal.pl*** script is run. The script needs to be run for each ChIP-Chip array experiment. The script takes several arguments, some required and one optional. The arguments are:

-c (Optional) If the configuration file is not the default geb_initialise.ini it needs to be declared. The file is required for the database settings, etc.

-s (Required) The species the array data is for (Human, Mouse etc)

-p (Required) The probeset name (For example “Nimblegen”)

-e (Required) The experiment name (For example “Exp_2”)

-u (Optional, default 1.4) The cut-off expression value for a probe to be included in the up-regulated histogram

-d (Optional, default 0.7) The cut-off expression value for a probe to be included in the down-regulated histogram

-t (Optional T/F, default T) Determines if the histogram for the probeset is created. This only needs to be created once. Default value is “T”, for subsequent experiments with the same probeset it can be set to “F”.

Run the script, with the required arguments, and the ChIP-Chip histogram data for the Java display will be created with the required cut-off values.
