# Supplementary material for: Genome Environment Browser (GEB): a dynamic browser for visualising high-throughput experimental data in the context of genome features
Source: BMC Bioinformatics. 2008 Nov 27;9:501. doi: 10.1186/1471-2105-9-501 (PMC2614995; doi:10.1186/1471-2105-9-501)
Supplement: Additional file 2 — GEB user guide. GEB tutorial with detailed description of each feature and functionality of the browser. [file 1471-2105-9-501-S2.pdf]

Genome Environment Browser (GEB) user guide

GEB is a Java application developed to provide a dynamic graphical interface to visualise the distribution of genome features and chromosome-wide experimental data in high resolution.

(I) Genome Features Annotated in GEB

The demonstration ("demo") version of GEB provides annotation for human (NCBI Build 36, Ensembl database version 50.36i) and mouse (NCBI Build 36, Ensembl database version 46.36g) genomes. GEB can display data from any genomes available at Ensembl if custom GEB databases have been built (please refer to the GEB installation guide). In each demo genome, the following standard features were annotated:

1. ***Genes***: Exon-intron location of protein-coding genes was obtained from Ensembl. Both Ensembl known and novel (predicted) genes were included. In addition, where a gene produces more than one transcript (e.g. through alternative splicing or alternative promoter usage), information for individual transcript is available.

2. ***Non-coding genes:*** Non-coding genes annotated by Ensembl. They include: pseudogenes (processed and unprocessed), tRNA (nuclear transfer RNA, or pseudogene), mt-tRNA (mitochondrially-derived tRNA pseudogenes located in nuclear genome), rRNA(ribosomal RNA or pseudogene), scRNA(small cytoplasmic RNAor pseudogene), snRNA (small nuclear RNA or pseudogene), snoRNA (small nucleolar RNA or pseudogene) and miRNA (microRNA precursors or pseudogene), misc_RNA (miscellaneous other RNA).

3. ***CpG islands***: The program newcpgreport (EMBOSS) was used to screen genome sequences (obtained from Ensembl) for CpG islands. Parameters for each CpG island were set to default: size of CpG island at least 200bp, C+G content at least 50% and observed CpG/expected CpG at least 0.6.

4. ***Repetitive elements***: Annotation for repeats was taken directly from Ensembl, which in turn adopted the RepeatMasker output. Three major types of repetitive elements were displayed: LINEs (long interspersed nuclear elements), LINE-1 (L1, being a subset of LINEs), SINEs (short interspersed nuclear elements) and LTRs (long terminal repeats). All other repetitive elements, such as low-complexity repeats and DNA transposons, were grouped under the “Other repeats” category.

To demonstrate GEB’s versatility, we have included the following examples of custom annotation of L1s in the human and mouse genomes. Each L1 element identified by RepeatMasker is known as a “match”. In GEB, each L1 match was further annotated as being the 5’ UTR, ORF1, ORF2 and 3’UTR, depending on where exactly the L1 match aligns to the L1 consensus sequence. Any L1 element which is 6kb or longer with no internal inversion was scored as a FL-L1.

(II) Configurating and launching GEB

Users are strongly recommended to review and edit (if required) the **geb.ini** configuration file ***prior to*** launching GEB as it defines the Ensembl database, species, genomics features, etc to be displayed on the Java viewer . A sample configuration file has been provided as a template and can be used to test GEB as it connects to a sample database at Imperial College.

Note: All settings in the ini file must be in lower case, except feature and repeat names.

Details of the configuration files:

- The first section of the **geb.ini** file defines the database to connect to.

  [database]
  host = localhost
  port = 3306
  username = guest
  password = guest

- The next section specifies the species to be accessible in the Java viewer.

  [species]
  mouse_46_36g = yes
  human_46_36h = no

  If set to ***no***, or omitted, then the specified species will not be available in the viewer.

- The next section specifies the species details.

  [mouse_46_36g]
  chromosomes = 21
  x = 20
  y = 21
  name = mus_musculus

- Next are the features to display, all of which must obviously be available in the relevant GEB database.

  [features_mouse_46_36g]
  Genes = 2
  Non_coding_genes = 2
  CpG = 1
  UTR5 = 2
  ORF1 = 2
  ORF2 = 2
  UTR3 = 2

  The number assigned to each feature specifies how may strands of the chromosome it is assigned to. CpG islands are strand neutral so the value is ***1***

meaning they are displayed only on one strand. All features that are assigned to both strands should be set to ***2***. If not, this can have a detrimental effect on the display.

- Next are the repeats, with the same strand designation.

[repeats_mouse_46_36g]
LINE/L1 = 2
LINE = 2
SINE = 2
LTR = 2
Other_repeats = 2

- One of the reasons for developing GEB was to allow the visualisation of features in 2 dimensions, something not supported by other browsers. It was a requirement that the length of features in the physical map display should be represented vertically, as well as horizontally, to give a clearer visualisation of their relative size. By default all features have a fixed vertical size but if this functionality is required then the optional “Lengths” section can be used to specify the relevant features. The number assigned is the overall maximum size for that feature. If the length setting is used it means that the relative size of a feature is clearly visible.

[lengths_Mouse_46_36g]
UTR5 = 1030
ORF1 = 1016
ORF2 = 3293
UTR3 = 2475

- The final species-specific entry is for the microarray data to display.

[microarray_mouse_46_36g]
expression = no
chip_chip = yes
chip_chip _pos = 1.4
chip_chip _neg = 0.7

If set to ***no***, or omitted, then the specified array type will not be available in the viewer.

- For the expression array data, the default values of the minimum/maximum expression values for the histogram display can be set. This can also be changed in the Java views. The ChIP-Chip min/max values are pre-set due to the large number of probes and any change here will not affect the histograms. By default they will be 1.4 and 0.7, but if different values were used when the microarray data was processed for GEB, then the correct values can be set here so the viewer shows the correct versions.

- The final setting is for the colours to be used for each feature. These colours will be used for all species displayed. The colours section is optional and if omitted, or individual features are omitted, colours will be dynamically assigned. Colour choices are green, red, blue, magenta, cyan, yellow, orange, grey, white and black.

[colours]
Genes = green
Non_coding_genes = green
CpG = magenta
LINE/L1 = yellow
LINE = orange
SINE = grey
LTR = white
Other_repeats = black

When all the settings have been reviewed, save the changes on the configuration file (if edited).

Launch GEB by double-clicking the **GEB.jar** file, or by typing on the command line: `java –jar GEB.jar`.

(III) Browsing Capabililities of GEB

******** Welcome Page for Displaying Standard/Custom Genomic Features ********

1. Select the species and chromosome of interest from the dropdown boxes. (Also note point no. 6 below)

2. "Range" controls the width of each histogram bar (the non-sliding counting window). Set at 1Mb by default, it can be changed to 500kb or 100kb for finer plots.

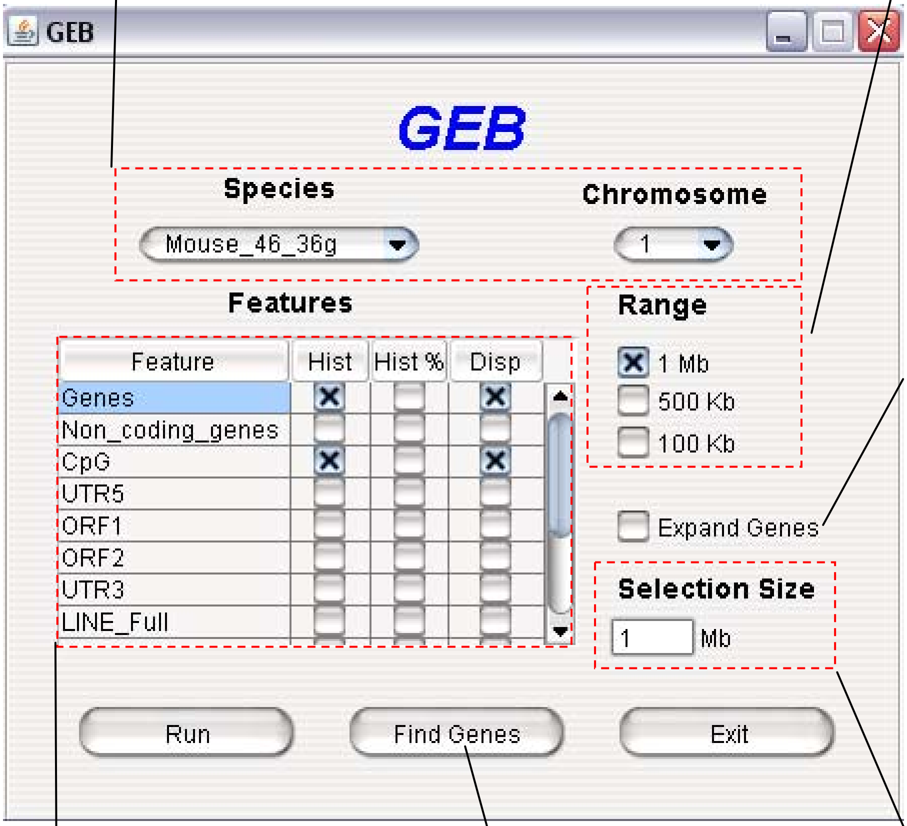

3. Select the genomic features to be displayed on histogram (Hist) and physical map display (Disp).

   "Hist": displays copy number of each feature in the range.

   "Hist%": displays the % of sequence contributed by each feature in the range.

4. "Expand genes" allows alternative transcripts for a given gene to be displayed in the physical map. Otherwise only the longest transcript will be shown.

5. "Selection Size" specifies the width of the blue selection bar used for panning across the chromosome-wide histogram. The default width of the bar is 1Mb and can be set to any value (in Mb).

6. Search your gene of interest by Ensembl gene ID/description. Once the gene is found, GEB will skip the histogram display and go straight to the physical map display for the gene with 1Mb flanking sequence (500kb either side). Note that "species", "features" and "Expand Genes" options still applies.

***** *Welcome Page Including Options for Displaying Microarray Data* *****

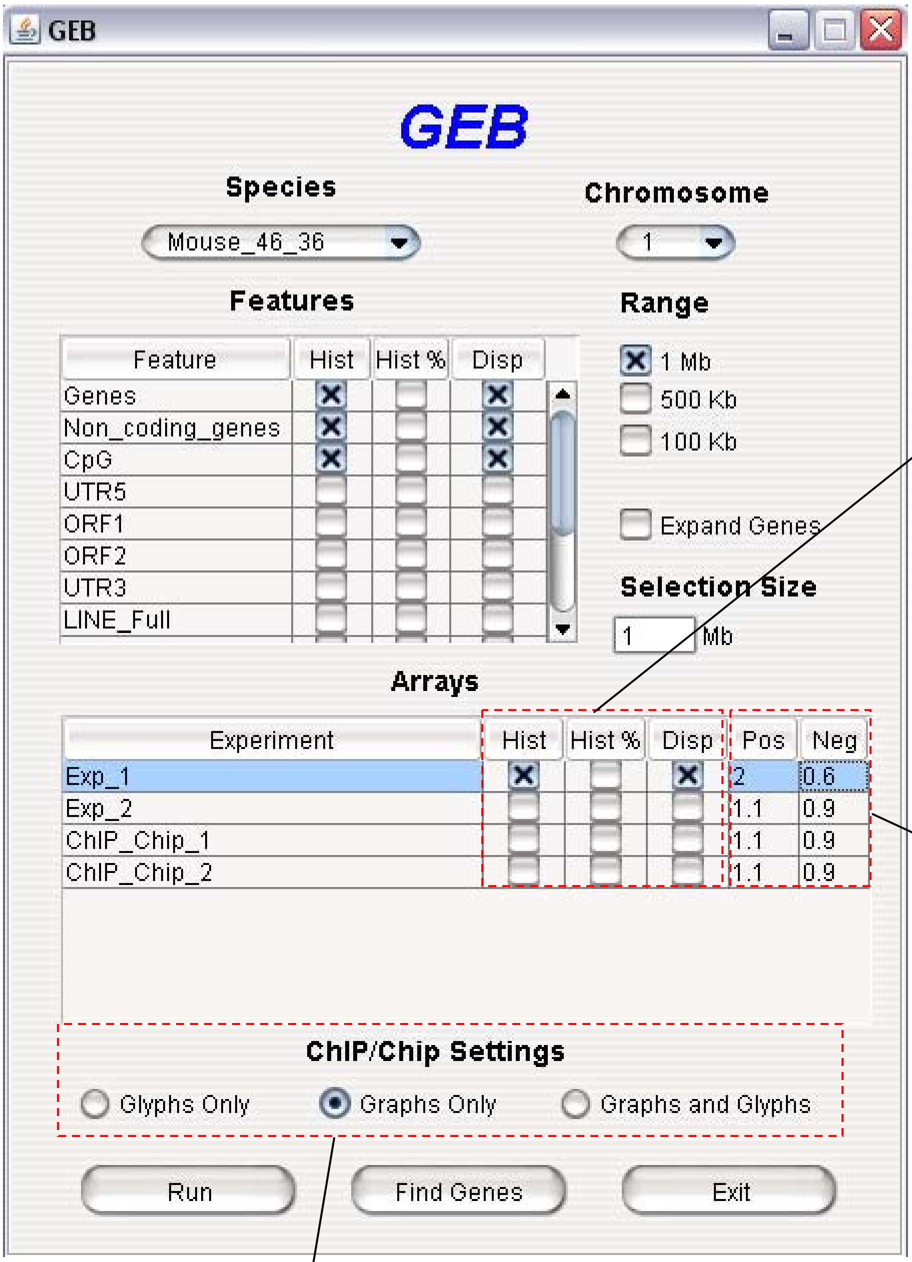

"Hist": displays copy number of each feature in the range.

"Hist%": displays the % of sequence contributed by each feature in the range.

"Disp": show data in the physical map display page.

Type in the required gene expression thresholds for the expression arrays here#.

For example, setting a "Pos" threshold of "2" will display genes with 2x expression relative to control (i.e. a "100%" increase or "2-fold" upregulation).

Likewise, setting a "Neg" threshold of "0.6" will display genes with 0.6x expression relative to control, (i.e. 40% decrease in gene expression).

Data display options for ChIP/chip or tiling array data.

Glyphs are best suited for viewing global patterns, while graphs are more suited for analysing local patterns. See examples on page 12 of this user guide.

#Thresholds for tiling arrays are hard-coded in the ***geb.ini*** configuration file and cannot be changed on the welcome page.

Important notes about the welcome page:

1. Closing the welcome page will automatically close *all* other GEB windows. Please make sure it remains opened when GEB is in use.

2. Histogram and physical map displays are constantly “listening” to the options selected on the welcome page. For example, a user at the beginning of the session might have selected to show “genes” only in the physical map display. Later, while browsing the histogram display page, the user might suddenly decide to display “CpG islands” too in the physical map display. In this case, the user can go back to the welcome page (which is always opened), check the “CpG” box for “Disp”, and then load the physical map display directly from the “original” histogram (which was loaded *before* the “CpG” option for “Disp” was selected). There is no need to “reload” the histogram in order for the “CpG on physical display” instruction to be executed.

3. In “Features”, if “non-coding genes” is not selected but “genes” is, then non-coding genes will be included in the “genes” track.

***** *Histogram Display - Panoramic View Across a Chromosome* *****

- As an example, the “range” (width of each histogram bar) is set at 1Mb.
- Histogram scale between different features is *not* standardised because of the huge variation in copy number between features.
- “Tools” is shared with the physical map display (see section IV).

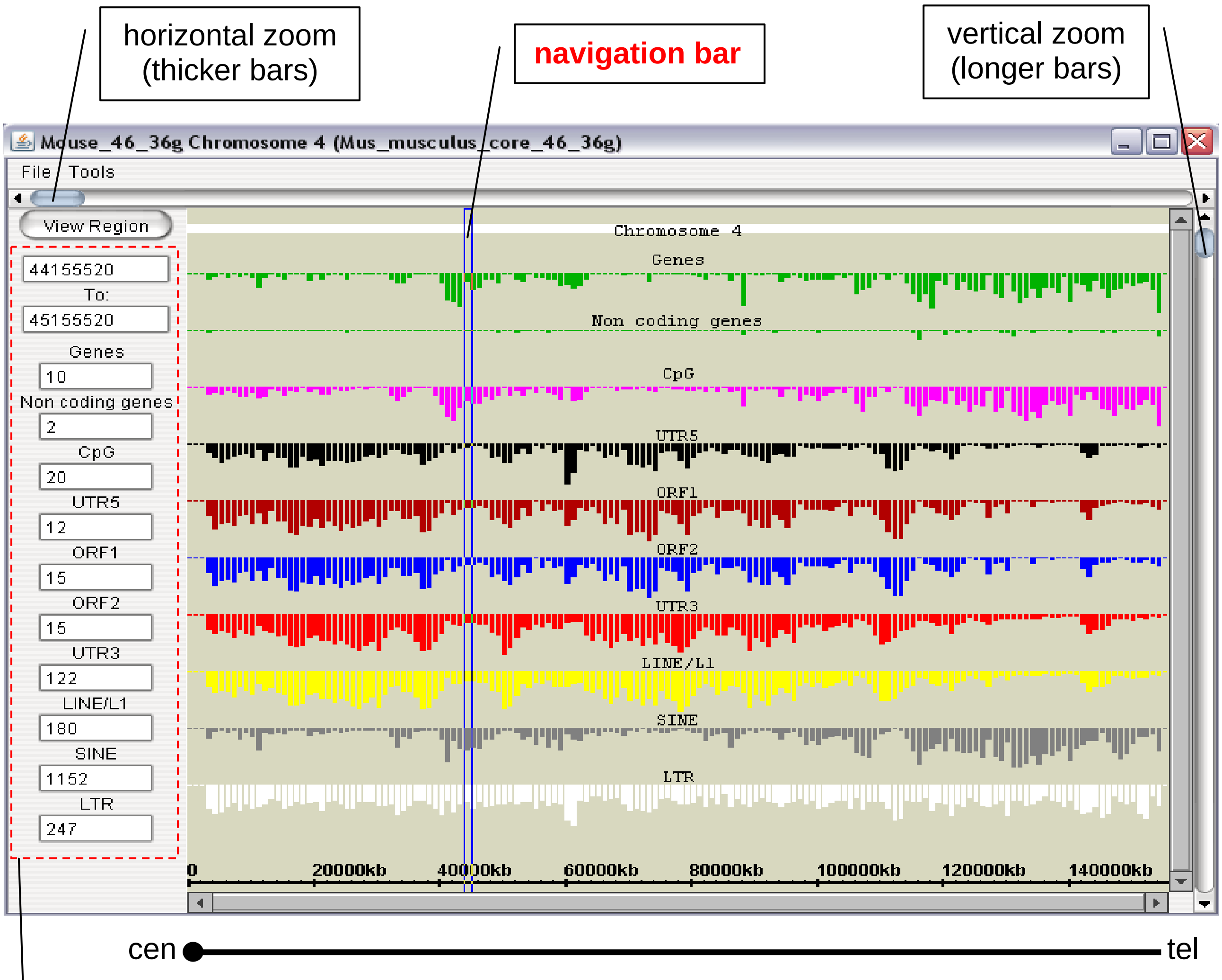

This panel displays information related to the genomic region selected by the navigation bar. Genomic coordinates can be set by the bar, or typed in manually. To navigate in “fixed” steps, e.g. 45-46, 46-47 and 47-48Mb instead of in “irregular” steps (e.g. 44155520-45155520 as above), choose “Fix Scroll” under tools.

Range of genomic coordinates allowed is 500bp-25Mb.

The copy number of each genomic feature in the proximal 1Mb interval is shown. In this example, the numbers correspond to the interval of 44-45Mb.

***** *Physical map display - detailed view of region of interest* *****

- All features are shown on both the sense and anti-sense strands (above and below the ruler respectively). Exons appear as green boxes, while introns appear as green lines in between exons.

- Detailed L1 annotation is shown here as an example of the flexible two-dimensional display interface.

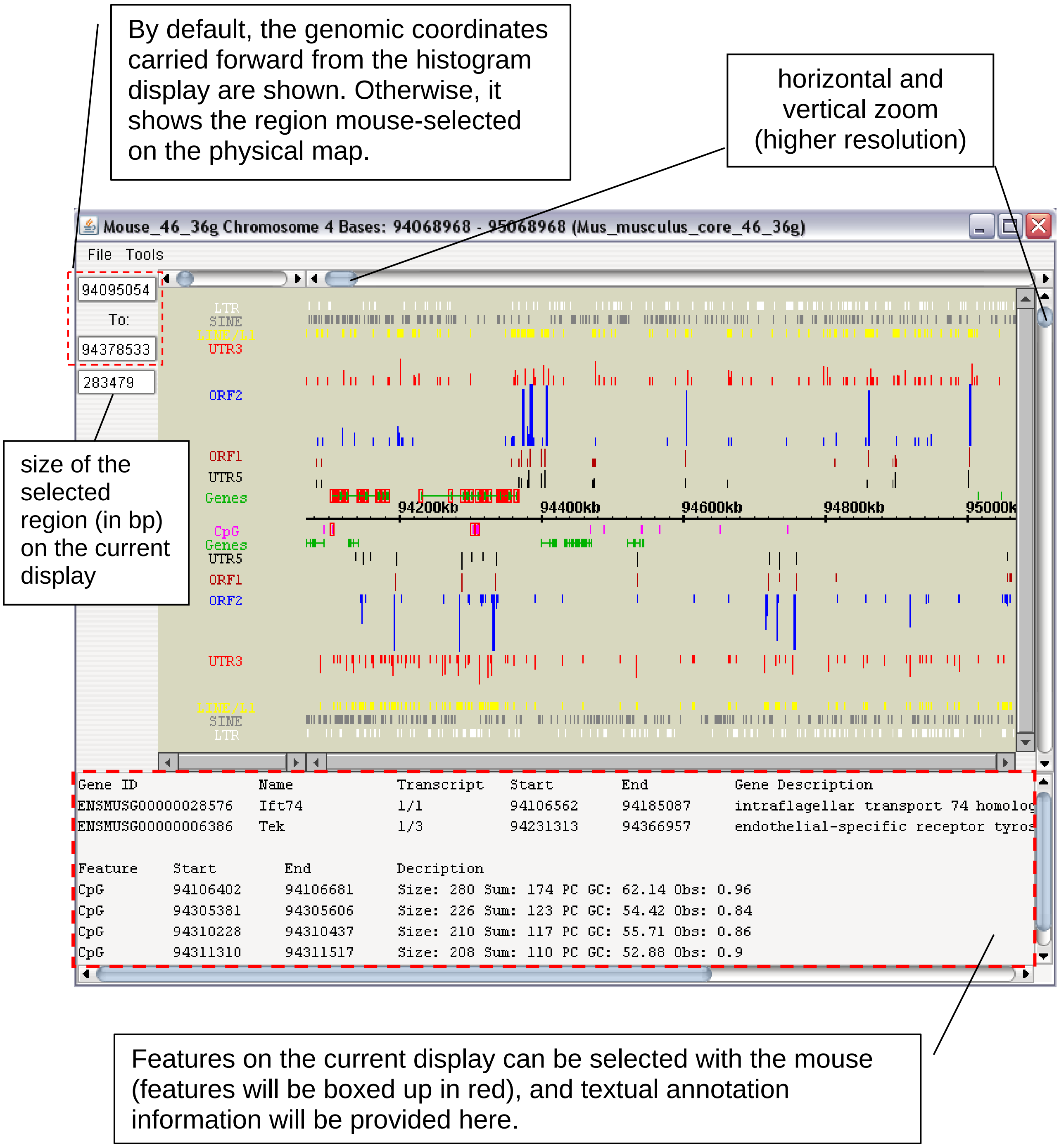

***** *Physical map display 2 - for gene expression microarray data* *****

- More than one gene expression array data sets can be displayed. In this example, two data sets have been selected. However, loading two or more data sets is not recommended if the “expand genes” option has been selected, as the display will become cluttered.

- Gene expression data and tiling array data *can* be displayed at the same time. (See tiling data display on page 12.)

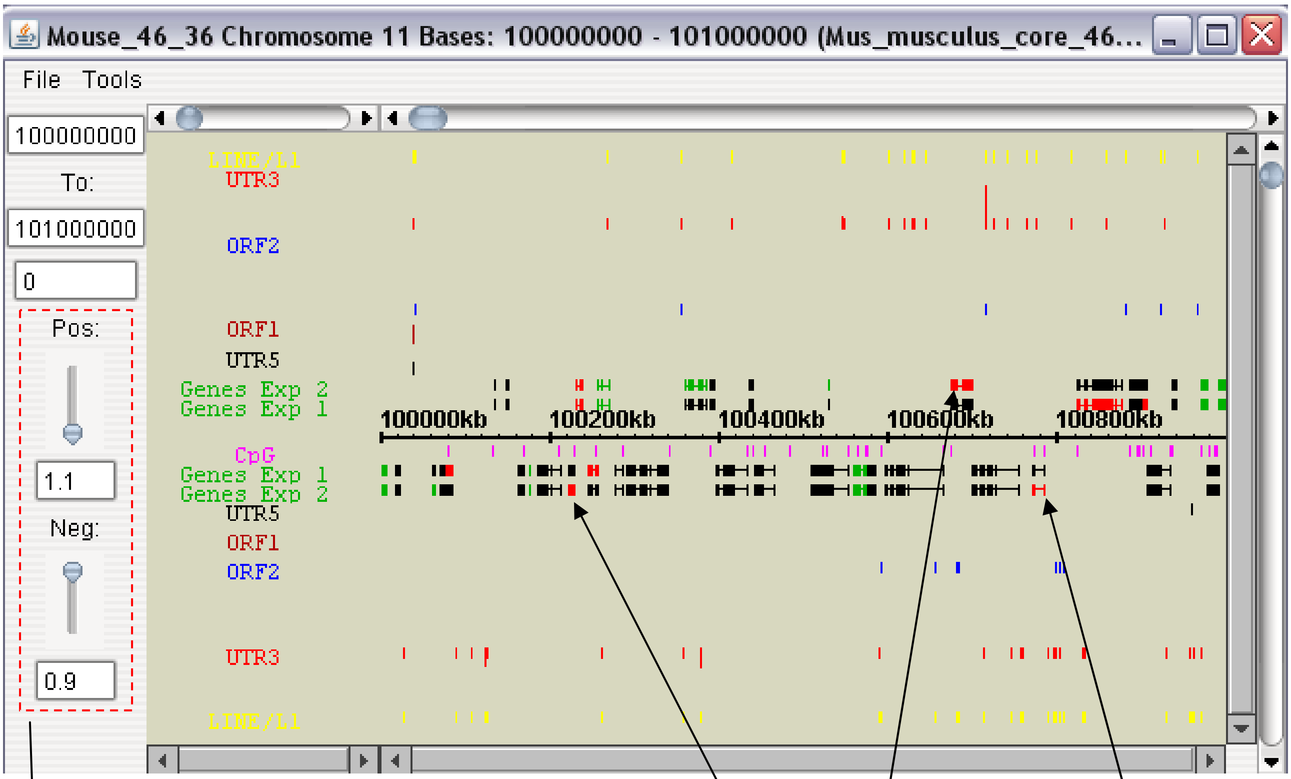

Sliding scales for real-time adjustment of gene expression threshold, “Pos” for upregulated genes, “Neg” for downregulated ones.

The initial values of the thresholds are set by the values entered on the welcome page.

Genes will be colour-coded according to these initial thresholds when the physical map display is first loaded.

Genes with no data remain green. Differentially expressed genes (DEGs) are coded red (upregulated), blue (downregulated) or black (no change in expression).

In this example, there are 5 upregulated genes in the dataset “Exp 1”.

As the thresholds are changed, DEGs which lose their status as differentially-expressed will turn black.

***** *Physical map display 3 - for tiling microarray data (glyphs)* *****

- As for gene expression data, more than one tiling array data sets can be displayed. It is not recommended to load too many data sets due to cluttering.

Glyphs: each glyph represents one probe. Probes are colour-coded to reflect their signal relative to the control sample(s):
red = enrichment; blue = depletion; black = no change.

Closely-packed glyphs over hundreds of kbs could appear as blocks of red/blue/black, revealing specific patterns.

Graph (below the glyphs): It plots the probes' signal intensities on the y-axis. More useful when the physical map is zoomed-in, i.e. in higher resolution. Refer to page 13 for details.

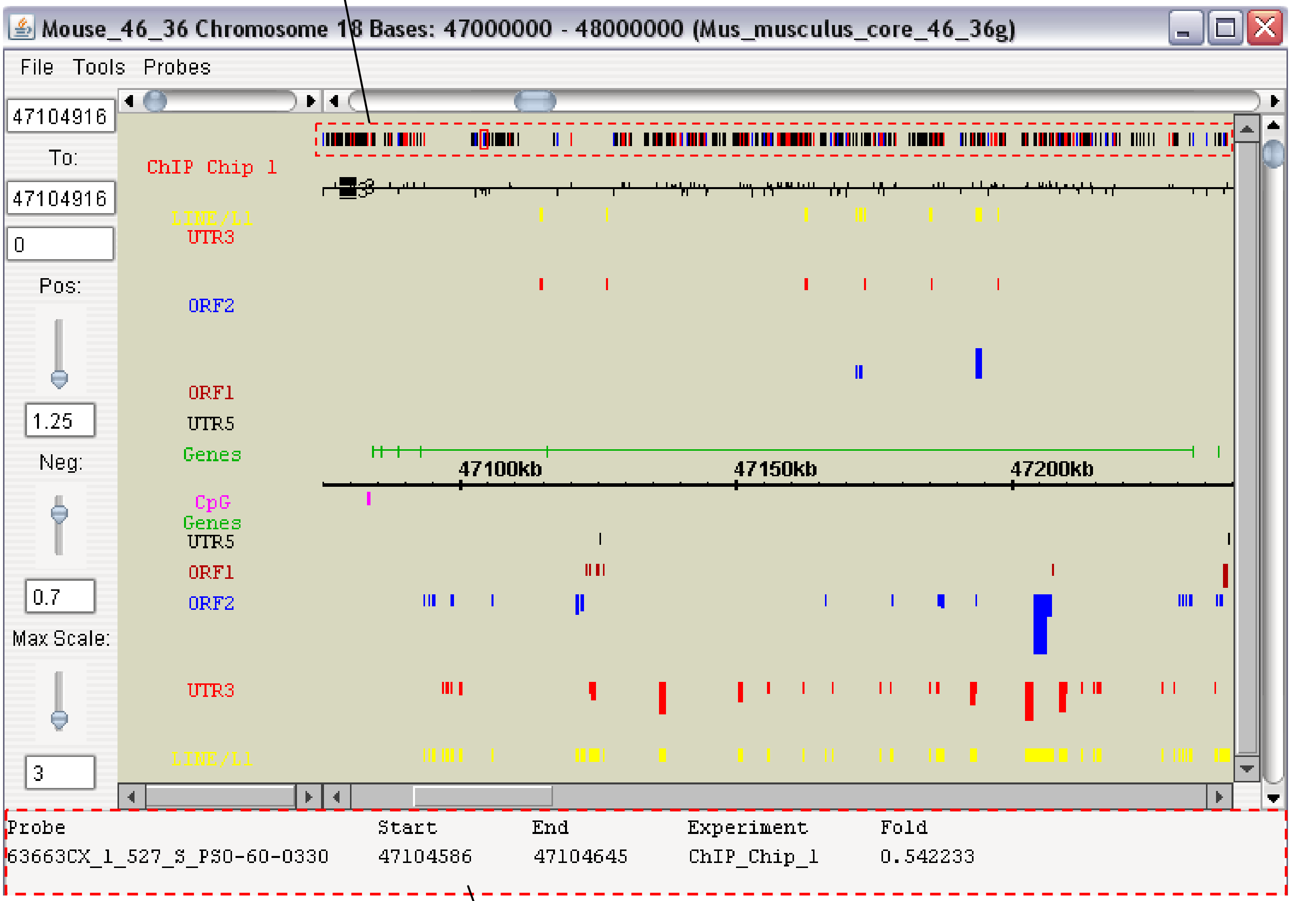

Refer to page 13 for details about the **sliding scales.**

As in other physical map display pages, each feature on the screen, e.g. a glyph/probe can be selected at a mouse click, and its related information will be displayed here.

***** *Physical map display 3 - for tiling microarray data (glyphs and graph)* *****

Check the required boxes here to display subsets of probes.

The “Probes” tab is available for glyphs, i.e. when “glyphs” or “graphs and glyphs” display is selected on the welcome page.

Graph: signal intensity of each probe is plotted on the y-axis. Peaks and troughs represent enrichment and depletion of signal with respect to control.

The graph is best observed when viewing the physical map display in high resolution. In this example, a 14kb region is shown.

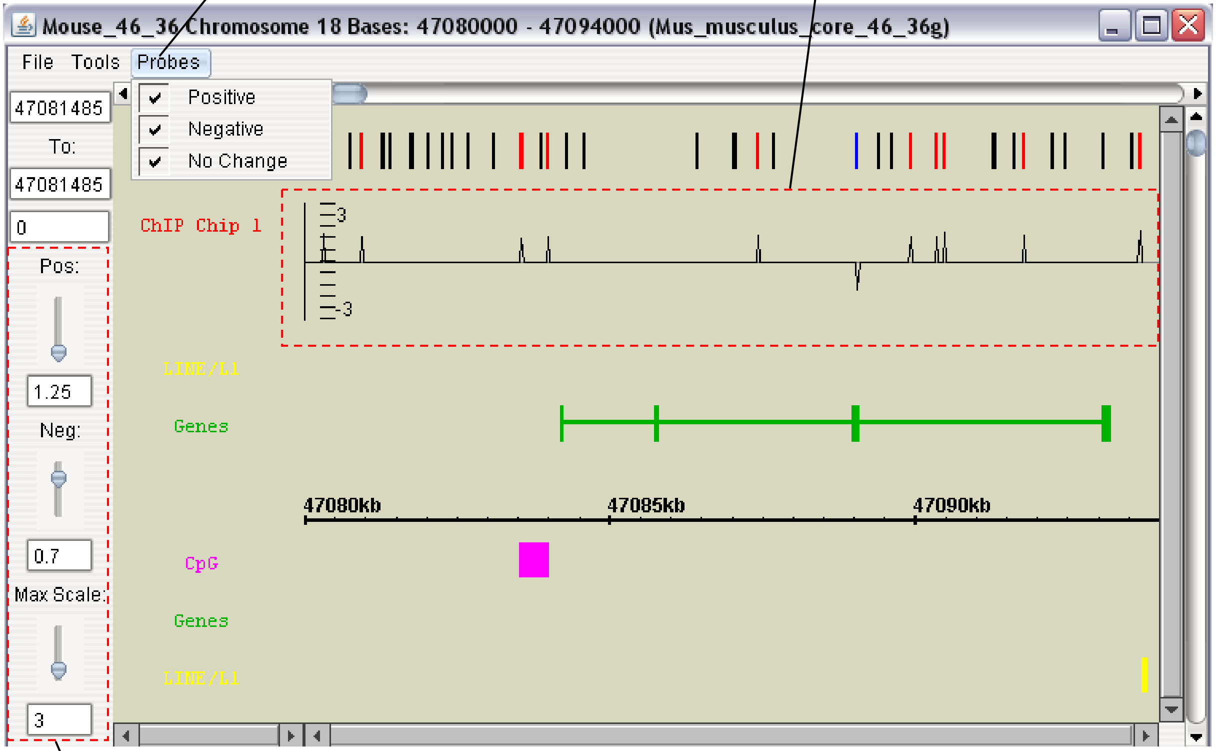

Sliding scales for adjusting the thresholds of signal enrichment or depletion in real-time. E.g. a “Pos” threshold of “3” means that only probes with at least 3x enriched signal intensity over control will be coloured in red. Probes below such threshold will be in black. Similarly, the “Neg” threshold sets the minimum depletion level required.

“Max Scale” bar is specific for graph display. It changes the scale of the y-axis dynamically.

(IV) GEB Tools

All the tools described in this section are available under the “Tools” tab at the top of the histogram and/or physical map displays.

1. ***Ensembl/Ensembl gene***: Because GEB is a browser specialised in graphical presentation of genomic features for visualisation, not all information about a genomic region or a gene is provided.

   Comprehensive information about a region (e.g. markers, DNA contigs, accessioned BAC clones, syntenic regions) can be obtained by the “Ensembl” function, which automatically triggers the user’s default web browser and links the region back to Ensembl ContigView via the base-pair position numbers. The position numbers are always determined by those entered/shown on the current screen from which the function is triggered.

   Similarly, extra information about a selected gene in the physical map display (e.g. orthologue prediction, transcript structure, SNPs) can be obtained by the “Ensembl gene” function, which automatically links the gene to its Ensembl GeneView page via its Ensembl gene ID.

2. ***Capture screen***: This function captures a snapshot of the display and saves it as a png file for printing and archiving.

3. ***View data***: This tool can calculate the copy number and/or percentage sequence representation of any type of annotated features across a genomic region of any size:

1. Select the **features** for which quantitative data are required.

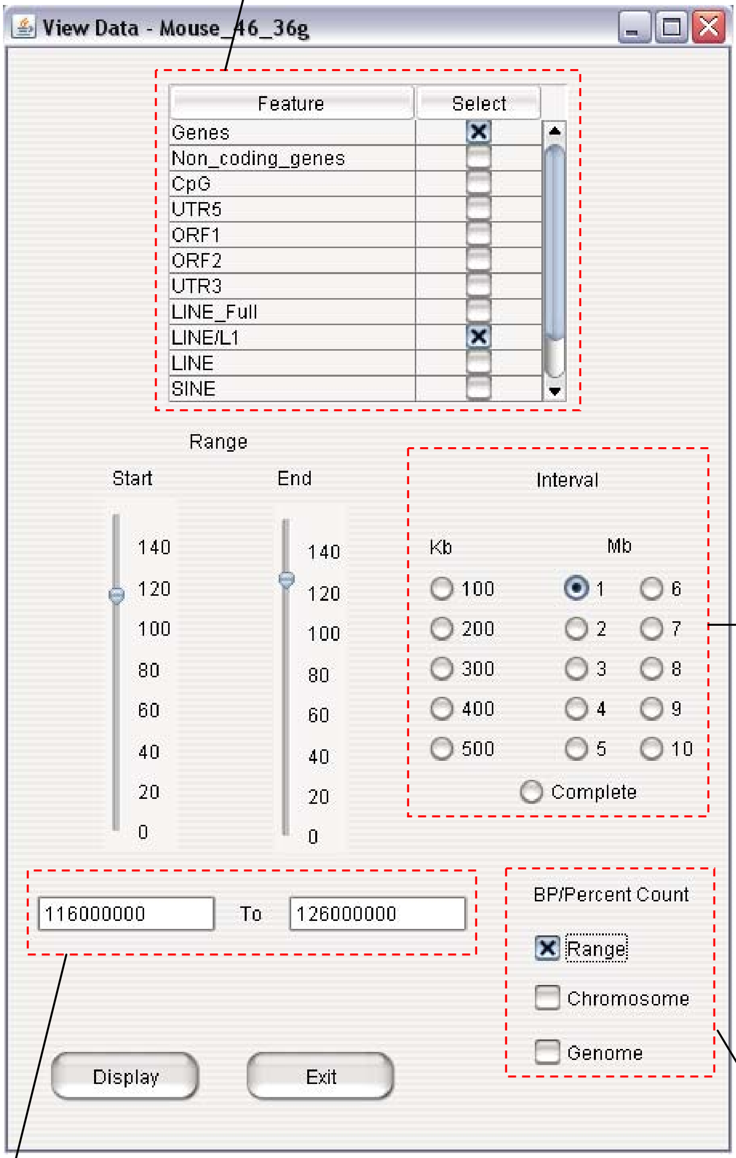

3. To analyse the region of interest in regular **intervals** (windows), select the required size.

   If the analysis should be done for the entire region without windows, select "complete".

4. The number of **basepairs** and **% sequence** contributed by each feature can be calculated.

   The percentage can be calculated with respect to the length of the range, the chromosome or the entire genome. These options are not mutually exclusive.

   If only the copy number of each feature is required, uncheck all boxes.

2. The genomic **range** over which the calculation will be done (region of interest).

   Coordinates are entered automatically from the screen from which "ViewData" was triggered. Can be changed manually by typing or by using the sliding scale above.
